# Supplementary material for: Non-invasive stress evaluation in domestic horses (Equus caballus): impact of housing conditions on sensory laterality and immunoglobulin A
Source: R Soc Open Sci. 2020 Feb 19;7(2):191994. doi: 10.1098/rsos.191994 (PMC7062079; doi:10.1098/rsos.191994)
Supplement: sampling schedule Marr et. al. 2020 (Table S1) [file rsos191994supp1.pdf]

FGM & faecal IgA sample collection time intervals

	-48 hours before start of test situation	-24 h	0 h	+24 h after start of test situation	+48 h	1 week	2 months	*No. data points averaged for statistical analysis*
Baseline (values taken before start of experiment)	x	x	x					*3*
(a) change from group housing to individual housing				x	x			*1/1*
(b) one week of individual stabling						x		*1*
(c) initial training				x	x			*1/1*
(d) two months regular training and individual stabling							x	*1*

motor & sensory laterality data collection time intervals

	60 observations for calcuation of laterality index
Baseline (values taken before start of experiment)	x
(a) change from group housing to individual housing	x
(b) one week of individual stabling	x
(c) initial training	x
(d) two months regular training and individual stabling	x
