# Supplementary material for: Non-invasive stress evaluation in domestic horses (Equus caballus): impact of housing conditions on sensory laterality and immunoglobulin A
Source: R Soc Open Sci. 2020 Feb 19;7(2):191994. doi: 10.1098/rsos.191994 (PMC7062079; doi:10.1098/rsos.191994)
Supplement: raw data: non-invasive stress evaluation in horses (Table S2) [file rsos191994supp2.pdf]

horse number	test situation	raw data FGM in ng/g	FGM difference between basal & test situation in ng/g	raw data IgA in µg/g	IgA difference between basal & test situation in µg/g
1	basal	24,47	0,00	9,33	0,00
2	basal	26,26	0,00	5,03	0,00
3	basal	38,26	0,00	14,11	0,00
4	basal	30,94	0,00	7,15	0,00
5	basal	65,68	0,00	9,11	0,00
6	basal	47,64	0,00	6,57	0,00
7	basal	36,33	0,00	12,64	0,00
8	basal	32,35	0,00	5,27	0,00
9	basal	56,52	0,00	7,47	0,00
10	basal	18,08	0,00	2,99	0,00
11	basal	32,67	0,00	6,17	0,00
1	a 24h	44,20	19,73	5,44	-3,89
2	a 24h	42,43	16,17	6,86	1,83
3	a 24h	46,52	8,27	4,08	-10,04
4	a 24h	43,92	12,98	13,87	6,72
5	a 24h	74,31	8,64	13,01	3,89
6	a 24h	50,30	2,66	1,91	-4,67
7	a 24h	58,96	22,64	14,05	1,40
8	a 24h	109,89	77,54	88,87	83,60
9	a 24h	88,95	32,44	6,28	-1,19
10	a 24h	36,80	18,72	4,02	1,03
11	a 24h	64,81	32,13	4,31	-1,86
1	a 48h	33,81	9,34	8,66	-0,67
2	a 48h	52,98	26,71	7,63	2,60
3	a 48h	30,85	-7,40	9,26	-4,85
4	a 48h	37,09	6,15	8,96	1,81
5	a 48h	58,80	-6,88	3,01	-6,11
6	a 48h	54,78	7,14	3,73	-2,84
7	a 48h	51,05	14,72	8,18	-4,47
8	a 48h	90,88	58,54	9,99	4,72
9	a 48h	79,91	23,40	6,80	-0,67
10	a 48h	53,92	35,84	9,41	6,42
11	a 48h	37,19	4,52	8,11	1,93
1	b	26,74	2,27	5,97	-3,36
2	b	30,13	3,86	4,14	-0,89
3	b	34,64	-3,61	5,39	-8,72
4	b	38,23	7,29	6,14	-1,01
5	b	63,92	-1,75	4,66	-4,45
6	b	33,55	-14,09	4,85	-1,73
7	b	32,77	-3,56	23,64	10,99
8	b	55,55	23,20	11,84	6,57

9	b	70,44	13,93	3,38	-4,09
10	b	43,27	25,19	8,42	5,43
11	b	44,51	11,83	5,46	-0,71
1	c 24h	33,39	8,92	4,25	-5,08
2	c 24h	52,49	26,23	2,95	-2,08
3	c 24h	29,84	-8,42	7,53	-6,58
4	c 24h	43,85	12,91	7,55	0,40
5	c 24h	45,31	-20,37	33,41	24,30
6	c 24h	64,07	16,43	3,13	-3,44
7	c 24h	53,50	17,18	12,60	-0,05
8	c 24h	46,20	13,85	2,67	-2,60
9	c 24h	82,93	26,41	2,05	-5,42
10	c 24h	48,49	30,41	3,87	0,88
11	c 24h	41,78	9,11	5,66	-0,51
1	c 48h	25,75	1,28	16,25	6,92
2	c 48h	42,20	15,94	6,15	1,12
3	c 48h	35,24	-3,02	19,96	5,85
4	c 48h	49,60	18,66	3,82	-3,33
5	c 48h	56,25	-9,43	6,03	-3,08
6	c 48h	47,46	-0,18	9,21	2,64
7	c 48h	49,07	12,74	7,62	-5,03
8	c 48h	71,60	39,25	4,00	-1,27
9	c 48h	113,34	56,82	5,75	-1,72
10	c 48h	41,10	23,02	9,70	6,71
11	c 48h	34,60	1,93	5,64	-0,53
1	d	43,79	19,33	11,38	2,05
2	d	40,83	14,57	5,09	0,06
3	d	33,19	-5,07	16,78	2,67
4	d	41,49	10,55	5,90	-1,25
5	d	60,09	-5,59	9,25	0,13
6	d	62,98	15,34	13,70	7,13
7	d	52,08	15,75	12,00	-0,65
8	d	65,49	33,14	10,11	4,84
9	d	72,50	15,98	2,32	-5,15
10	d	37,04	18,97	16,38	13,39
11	d	43,84	11,17	14,08	7,91

(a) change from group housing to individual housing
(b) one week of individual stabling
(c) initial training
(d) two months regular training and individual stabling

horse number	test situation	sensory laterality index	sensory laterality index difference between basal & test situation	motor laterality index	motor laterality index difference between basal & test situation
1	basal	0,71	0,00	0,10	0,00
2	basal	0,56	0,00	0,07	0,00
3	basal	0,14	0,00	0,10	0,00
4	basal	-0,05	0,00	0,40	0,00
5	basal	-0,09	0,00	0,00	0,00
6	basal	-0,43	0,00	0,37	0,00
7	basal	0,00	0,00	-0,07	0,00
8	basal	-0,09	0,00	-0,07	0,00
9	basal	0,00	0,00	0,00	0,00
10	basal	-0,18	0,00	0,03	0,00
11	basal	0,20	0,00	0,03	0,00
1	a	-0,71	-1,43	-0,37	-0,47
2	a	-0,60	-1,16	0,17	0,10
3	a	-0,20	-0,34	-0,07	-0,17
4	a	-0,25	-0,20	-0,30	-0,70
5	a	0,00	0,09	-0,13	-0,13
6	a	-0,82	-0,39	0,43	0,07
7	a	-0,80	-0,80	-0,17	-0,10
8	a	0,22	0,31	0,37	0,43
9	a	-0,53	-0,53	0,27	0,27
10	a	-0,14	0,03	-0,40	-0,43
11	a	-0,43	-0,63	-0,07	-0,10
1	b	-0,54	-1,25	0,00	-0,10
2	b	-0,57	-1,13	-0,17	-0,23
3	b	0,11	-0,03	-0,27	-0,37
4	b	-0,43	-0,38	0,17	-0,23
5	b	0,18	0,27	-0,27	-0,27
6	b	-0,22	0,21	-0,07	-0,43
7	b	-0,67	-0,67	-0,30	-0,23
8	b	0,25	0,34	-0,03	0,03
9	b	-0,27	-0,27	-0,03	-0,03
10	b	-0,50	-0,32	-0,53	-0,57
11	b	-0,14	-0,34	-0,30	-0,33
1	c	0,33	-0,38	-0,13	-0,23
2	c	-0,50	-1,06	0,07	0,00
3	c	-0,33	-0,48	-0,20	-0,30
4	c	-0,78	-0,73	0,10	-0,30
5	c	1,00	1,09	-0,10	-0,10
6	c	-0,30	0,12	-0,07	-0,43
7	c	-0,50	-0,50	-0,17	-0,10
8	c	-0,25	-0,16	-0,07	0,00

9	c	-0,50	-0,50	-0,13	-0,13
10	c	-0,45	-0,28	-0,13	-0,17
11	c	-0,20	-0,40	0,03	0,00
1	d	0,33	-0,38	-0,43	-0,53
2	d	-0,11	-0,67	-0,13	-0,20
3	d	-0,67	-0,81	-0,10	-0,20
4	d	-0,75	-0,70	0,27	-0,13
5	d	-0,25	-0,16	-0,35	-0,35
6	d	0,08	0,51	-0,10	-0,47
7	d	-0,50	-0,50	-0,57	-0,50
8	d	0,00	0,09	0,27	0,33
9	d	-0,33	-0,33	0,10	0,10
10	d	-0,33	-0,16	-0,73	-0,77
11	d	0,14	-0,06	0,17	0,13

(a) change from group housing to individual housing
(b) one week of individual stabling
(c) initial training
(d) two months regular training and individual stabling
